# Violent repression of environmental protests

**DOI:** 10.1186/s40064-016-1816-2

**Published:** 2016-02-29

**Authors:** Helen M. Poulos, Mary Alice Haddad

**Affiliations:** College of the Environment, Wesleyan University, 284 High St., Middletown, CT 06451 USA; Government Department, Wesleyan University, Middletown, CT 06451 USA

## Abstract

As global sea levels and natural resource demands rise, people around the world are increasingly protesting environmental threats to their lives and livelihoods. What are the conditions under which these peaceful environmental protests are violently repressed? This paper uses the random forest algorithm to conduct an event analysis of grassroots environmental protests around the world. Utilizing a database of 175 grassroots environmental protests, we found that: (1) a large proportion (37 %) of the protests involved violent repression; (2) most of the violence (56 %) was directed against marginalized groups; and (3) violence was geographically concentrated the global south in Latin America and Asia. The primary predictors of violence were political empowerment, GDP per capita, industry type, the presence of marginalized groups, and geographic region. Our analysis reveals a complex relationship between governance, resource extraction, and international funding that often resulted in human rights violations against marginalized groups.

## Background

Grassroots environmental protests represent a key mechanism for local populations to resist the negative effects of anthropogenic environmental hazards. However, these peaceful protests are sometimes repressed using violent means. The goal of this article is to identify the conditions under which peaceful environmental protests are more likely to be violently repressed and generate policy recommendations that could reduce or eliminate that violence.

Previous research has identified a number of factors related to the identity of the protesters, the content and duration of the protest, and its socio-political context that can affect the probability of violent repression. Numerous studies about Not In My Back Yard (NIMBY) politics have found that states and corporations commonly target vulnerable communities as site locations for environmentally hazardous projects, so those types of communities are more likely to be engaged in environmental protests and more likely to be violently repressed than better-resourced communities in the same countries (Adeola 2000; McGurty 1997; Rabe 1994; Roberts and Ash 2009). Scholars have also found that extractive industry projects are more often associated with violent repression than other types of industries (Downey et al. 2010; Blanton and Blanton 2009). Researchers have found when the two factors combine, when communities containing marginalized groups (defined herein as ethnic minorities or economically disadvantaged groups) protest natural resource extraction, they are particularly likely to be violently repressed (Koopmans and Rucht 2002; Bernauer et al. 2012; Homer-Dixon 1994). Scholars have also found that protest duration (Box-Steffensmeier et al. 2003) and the presence of documented human health hazards on site (Zheng and Liu 2013; Fagin 2013) can influence the likelihood of violent repression. Some scholars have found that violent repression of peaceful protests is more common in poor, authoritarian states that lack adequate legal protections than it is in rich, democratic countries with robust legal systems (Poe et al. 1999; Weede 1987; Besley and Persson 2011).

Thus far, research on violent repression of peaceful environmental protests has commonly used a case study methodology, which can help develop theories about the conditions that might result in violence against peaceful protesters, but cannot test those theories (Adeola 2000; Roberts and Ash 2009; Downey et al. 2010). Large-n research about violent repression has tended to analyze conflicts at the national level rather than local conflicts. While it is sometimes the case that grassroots environmental protests can transform into or combine with regime-threatening social movements (Haddad 2014), that outcome is unusual; locally-focused protests are much more common. Event analysis focusing on grassroots environmental protests has the potential to explain the interaction of subnational, national, and international factors related to the violent repression of peaceful protests (Bond et al. 2003; Box-Steffensmeier and Zorn 2002; Koopmans and Rucht 2002).

No prior research has employed a large-n statistical analysis to identify which factors and factor combinations are the most important in determining the likelihood that a grassroots environmental protest will be violently repressed. We employ the random forest algorithm using a suite of key violent repression predictor variables from the literature to examine the mechanisms underscoring violent repression in grassroots environmental protests using a database of environmental protests from around the world. We then use those results to develop a series of policy recommendations intended to reduce the rate and intensity of violence directed toward peaceful protesters.

## Methods

### Environmental protest database

To investigate the factors influencing violent outcomes in environmental protests, we compiled a global database of 175 grassroots environmental protest cases from a pool of 35,472 periodical publication search results from 1965 to 2013 using the Lexis-Nexis Academic (www.lexis-nexis.com) and Factiva (http://global.factiva.com) media databases. Factiva continuously archives periodical data from more than 32,000 sources from nearly every country worldwide in 28 languages, including over 450 continuously updated newswires (sources can be accessed here: http://www.proquest.com/documents/Title_List_-_Factiva.html?docID=242334851). Lexis-Nexis continuously archives news information from over 10,000 media sources (sources listed here: http://amdev.net/rpt_download.php). Together, they represent the most comprehensive databases of media coverage available. Media publications are useful because they provide information about large numbers of events and they are well-known for documenting social movement dynamics (Earl et al. 2004). While such event data can suffer from coverage bias (Ortiz et al. 2005; Barranco and Wisler 1999), they remain one of the only sources of data on protest events. To minimize biases and accuracy issues resulting from our use of a media-derived dataset, we: (1) searched for articles using multiple media database archives, (2) only included cases that were covered by more than one media source, and (3) confirmed and supplemented the information reported in the articles using ancillary information obtained through online sources (i.e. a series of web searches using Google to confirm separately that the event happened and to identify additional details about each protest event that were coded in the database as listed in Table 1). Ancillary data searches were performed as a means for completely coding the database because not all information in the database was directly stated in the short newspaper articles. If we could not find complete information to code the case completely in the database, we did not include the case in the study. Information coded using web information was included only if it was reported in more than one online search result. This case search methodology likely overlooked some environmental protest events that were suppressed by local governments and not reported by newspapers. For example, our sample size in the Middle East was quite low, probably because of poor reporting of protest events. Yet, even working within those limitations, the two databases we selected have the best world coverage of news events available, and they likely provided a representative sample of violent repression of grassroots environmental protests at regional- and global-scales.

**Table 1:** Variables

Violence	Binary variable, coded yes if there was any kind of physical violence ranging from beatings to death
Empowerment	CIRI Index’s New Empowerment Rights Index^a^
GDP per capita	World Bank Data^b^
Industry protested against	Chemical industry, development (construction or land clearing), hydroelectric, logging, mining, nuclear power, nuclear weapons, water, petroleum, waste disposal, or wind energy
Geographic region	Africa
	Central Asia
	Europe
	Latin America
	Middle East
	North America
	Oceania
	Southeast Asia
	Southern Asia
	Taken from United Nations (2012)
Project funded with international money	Coded yes if there was any international funding (e.g., multinational corporate involvement or international aid)
Protest duration	Number of years
Documented human health hazards of surrounding community	Yes or no
Governance	Free, partly free, or not free
Involvement of marginalized groups (defined as economically, socially, and politically disempowered people)	Yes or no

^a^CIRI data and documentation can be found here: http://www.humanrightsdata.com

^b^World Bank Data can be found at: http://data.worldbank.org/indicator/NY.GDP.PCAP.CD

We searched Lexis-Nexis and Factiva databases for a series of keywords associated with grassroots environmental protest based on a topic search of the environmental politics literature (Snow et al. 2008; Fillieule and Jiménez 2003; Dalton et al. 2003; Koopmans and Rucht 2002), which returned a total of 35,472 articles, which we scanned individually for grassroots environmental protest event data to build the final 175 case database. Search terms included: ‘grassroots environmental protest’ and the word ‘protest’ in conjunction with the terms ‘nuclear’, ‘pollution’, ‘conservation’, ‘environmental’, ‘wildlife preserve’, ‘toxic waste,’ ‘land fill,’ ‘mining,’ ‘dam,’ ‘hydroelectric,’ and ‘wind energy’. Protests were excluded from the study if they (1) failed to demonstrate evidence of locally-generated activism (i.e. grassroots), (2) lacked sufficient qualitative information about protest characteristics, or (3) appeared in fewer than two newspaper articles. Cases were then categorized into two groups: protests that remained nonviolent throughout and cases where some form of violence against activists occurred. Violence included any form of injury ranging from beatings to death.

The potential explanatory variables included in this study were chosen based on their prevalence in the newspaper articles and their prior identification as important predictors of environmental protest success by other prior researchers (Ash 2011; Aldrich 2008; Rabe 1994; McGurty 1997). The authors were entirely responsible for coding the database. We attempted to reduce coding biases by taking a random sample of 25 % of the study cases and performing reciprocal recoding as a reliability test, which resulted in no significant changes to the database based on a general linear model analysis of the changes in variable frequency (*P* > 0.001).

For each protest case, newspaper articles were gleaned for information on potentially important attributes that could play roles in violent outbreaks based on our review of the literature. For each case, we used the rubric in Table 1 to code each case for a range of characteristics that could contribute to violent episodes in environmental protests. Each case contained full attribute records for each potential predictor variable, which restricted our full dataset to 175 cases.

We coded the dependent variable, violence, as a binary variable. If protests were peaceful and authorities used peaceful, lawful means of managing and dispersing the protests, the event was coded as not violent. If there was any physical violence at all against protesters, ranging from roughing up to beatings and murder, the protest was coded as violent.

Regime type was determined using Freedom House’s Freedom in the World report, which rates countries as free, partly free, and not free according to a number of measures related to political rights and civil liberties. A number of indices that evaluate democratic freedoms on a country-by-country basis, yet most of them remain highly correlated (Zheng and Liu 2013). We selected the Freedom House Database because of its wide use in scholarship and its long record of historical data, which begin in 1972 (Freedom House 2013) and allowed us to code for democratic freedoms over the exact years of all of the cases included in our study.

To gain a more nuanced measure of state propensity toward repression we also utilized the CIRI Human Rights Dataset. This dataset has been widely used in human right research (Cingranelli and Richards 2010). From this dataset we utilized the empowerment variable that measures government respect for five human rights (movement, speech, worker’s rights, political participation, and freedom of religion). The CIRI empowerment index was coded using the mean values over the duration of each protest event. Since the CIRI empowerment index includes measures of freedom of speech, it also helps mitigate our study against the possibility that our measures of violence against protesters are actually measuring a greater willingness of the press to report protests/violence rather than greater incidence of protest/violence. While other variables in the CIRI dataset, such as the Physical Integrity Rights Index or Torture, might have been appropriate in theoretical terms, it is likely that the events of violence against protesters collected in our dataset would also have been picked up by the PIRI measure, creating an endogeneity problem.^1^

GDP per capita was measured using World Bank Data (data.worldbank.org) using the same coding methodology as employed in the CIRI empowerment index (i.e. mean values over the duration of each protest event). We also coded the industry that was primarily related to the protest: chemical industry, hydroelectric, logging, mining, nuclear power, petroleum, waste disposal, or wind energy. We had an additional category of development that we coded within the industry variable to cover the handful of cases that could not be attributed to a particular industry and involved large construction projects, usually highways but also bridges, railways, and tourist resorts.

Marginalized groups were coded as positive if the conflict involved any marginalized groups. Marginalized groups were always economically, socially, and politically disempowered people. In most cases they were also ethnic minorities. Geographic region was coded using the United Nations geographic designations (United Nations 2012). International funding was coded as positive if there was evidence of international money involved in the issue under protest (multinational corporations were the most common source; occasionally projects were funded through international funding organizations such as the World Bank). The protest duration was measured as the number of years from the start of the protest to its conclusion. If the conflict continued to the present, the duration of the protest was measured from the start of the protest to 2013, when we collected the data. We also coded for the documented presence of human health hazards in the protest events because the prevalence of human health hazards at a site can serve as a catalyst for grassroots protest (Zheng and Liu 2013; Fagin 2013). Human health hazards were coded as positive if we could find scientific evidence that there was a human health hazard related to the specific conflict event.

### Statistical analysis

All statistical analyses were performed using the R Statistical Language using the party package (R Development Core Team 2015). We used random forests (RF) (Breiman 2001; R Development Core Team 2015) to identify explanatory protest characteristics that differentiated cases where grassroots environmental protests were violently repressed and cases that remained nonviolent throughout. The model incorporated a total of 9 potential predictor variables to differentiate the protest cases into violent or non-violent groups.

The RF classification algorithm is an extension of the classification and regression trees (CART) (Breiman 1984). Classification and regression trees have been widely used to group two or more known classes of observations based on a suite of predictor variables. Classification using CART is achieved through recursive partitioning of the dataset into successively more homogeneous groups. The results are homogeneous subsets of the data based on a series of splits using all of the predictor variables, where the best tree structure is determined by the Gini Index. We used the cforest function in the party package to build our random forest model which using conditional permutation importance. Random forest in this implementation produces multiple CART-like tree classifiers, each based on sub-sampling without replacement (Hothorn et al. 2006). The advantage of using the ctree function in the party package over the original random forest implementation by Breiman (2001) is that it produces unbiased individual trees.

We built a random forest model of 10,000 trees to estimate the predictor variable importance for violent and nonviolent environmental protests. The random number of predictor variables included in each split (mtry) was three which was the square root of the total number of predictor variables as recommended by Breiman (Strobl et al. 2008). We also performed a sensitivity analysis for different numbers of trees and mtry values, but few changes in model output occurred for mtry ranging from 2 to 10, so the final model used the mtry default value of three and 10,000 trees since computation time was not a hindrance to model construction. Informative predictor variables were determined following Strobl et al. (2008) who indicated that can be considered informative and important if their variable importance value is above the absolute value of the lowest negative-scoring variable. We also estimated various model performance parameters in addition to the standard variable importance measures produced including overall model accuracy, the Kappa Statistic, and the area under the curve (AUC) of the receiver operator characteristic.

## Results and discussion

The following section discusses each of the variables in the study separately and highlights interactive effects. The most powerful predictors of violent repression of environmental protesters were low levels of civil liberties and low economic development. The duration of the protest was not a significant predictor of violence, which suggests that violent repression can occur in both intense, short-duration protests, as well as in long, multi-year environmental protests. Industry type was also significant; industries involving natural resource extraction elicited significantly more violence than other industries.

There were complex interactions in the relationships among regime type, presence of marginalized groups, geographic region, and presence of international funding and the use of violence against environmental protesters. Marginalized groups were disproportionately victims of violence around the world, particularly in democratic countries. The most intense cases of violent repression (cases with five or more deaths) all involved extractive industries. Nearly all of them (nine of ten cases) involved international funding, and nine of ten cases also involved marginalized groups.

The random forest model accuracy assessment resulted in a robust model output. The overall model accuracy was 86.8 %. The AUC was 0.94, and the model had a kappa statistic of 0.72. These figures indicate good overall model performance.

**Fig. 1:**
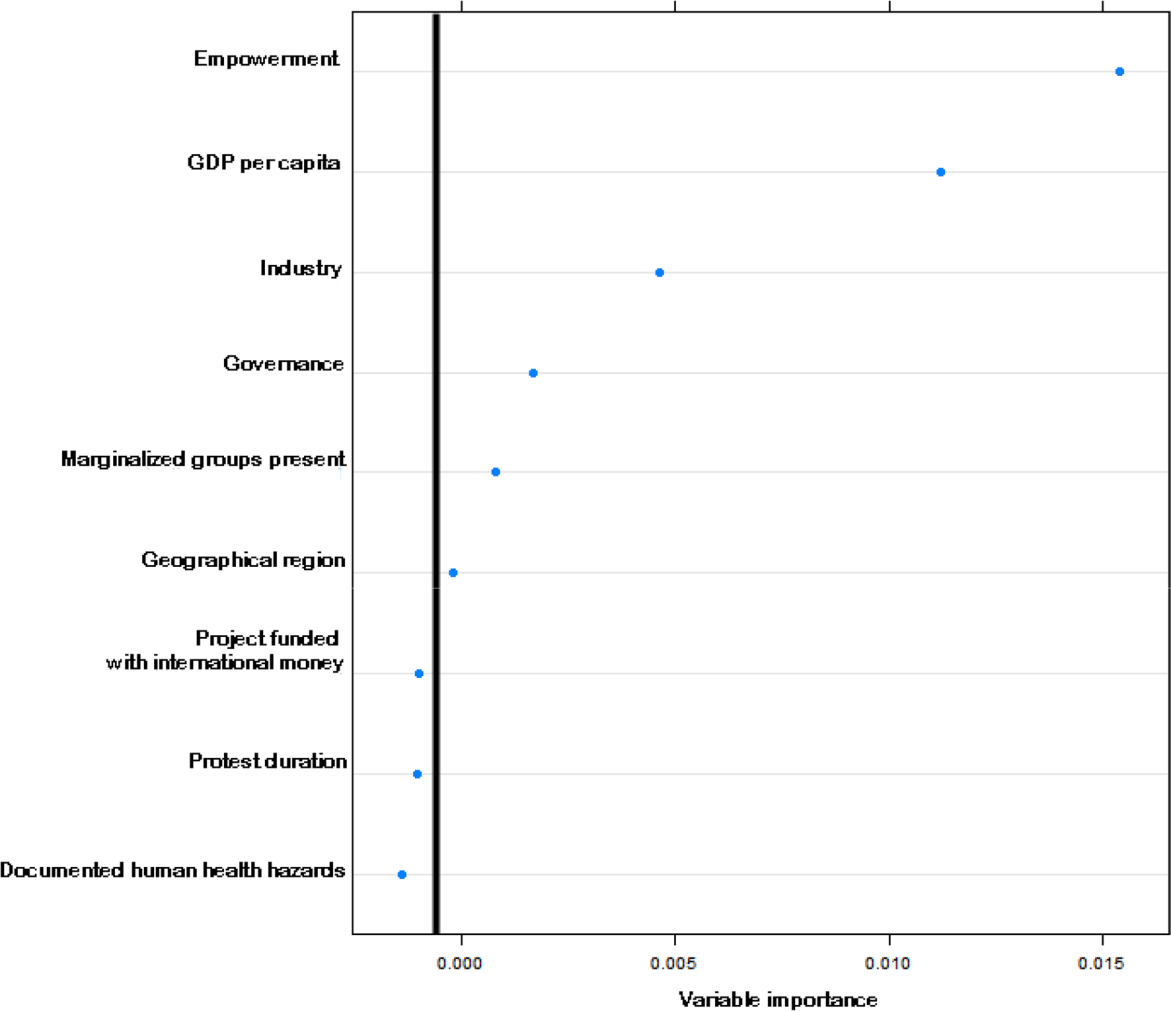
Factors influencing the violent repression of environmental protests. Figure lists the importance of protest characteristics that differentiated violent repression of grassroots environmental protests from nonviolent responses. The significance cutoff for informative explanatory variables is indicated by the *vertical line*, which represents the value above the absolute value of the lowest negative-scoring variable. Variable descriptions are provided in Table 1

The CIRI empowerment index was by far the best predictor of violent repression against grassroots environmental protests (Fig. 1), which fits our expectations that countries with greater civil rights general have lower incidences of violent repression against protesters. Empowerment was significantly lower (*P* < 0.0001, *t* test) in countries with violent repression than in countries where violence did not occur. This finding supports the voluminous research using cross-national data, which has shown that protection of basic civil liberties inhibits human rights abuse (Cross 1999; Hill and Rothchild 1992; Keith et al. 2009).

As expected, countries with higher levels of economic development (as quantified by average GDP per capita) repressed citizens less than those with lower levels of development ($21,940 and $8875, respectively). This is not to say that violence did not occur in more developed countries, but rather that there was a general trend of less violent repression in more economically affluent geographical locations. Once again, this finding supports numerous studies that have demonstrated the connection between higher levels of GDP per capita and lower levels of governmental repression (Besley and Persson 2011; Weede 1987; Henderson 1991).

**Fig. 2:**
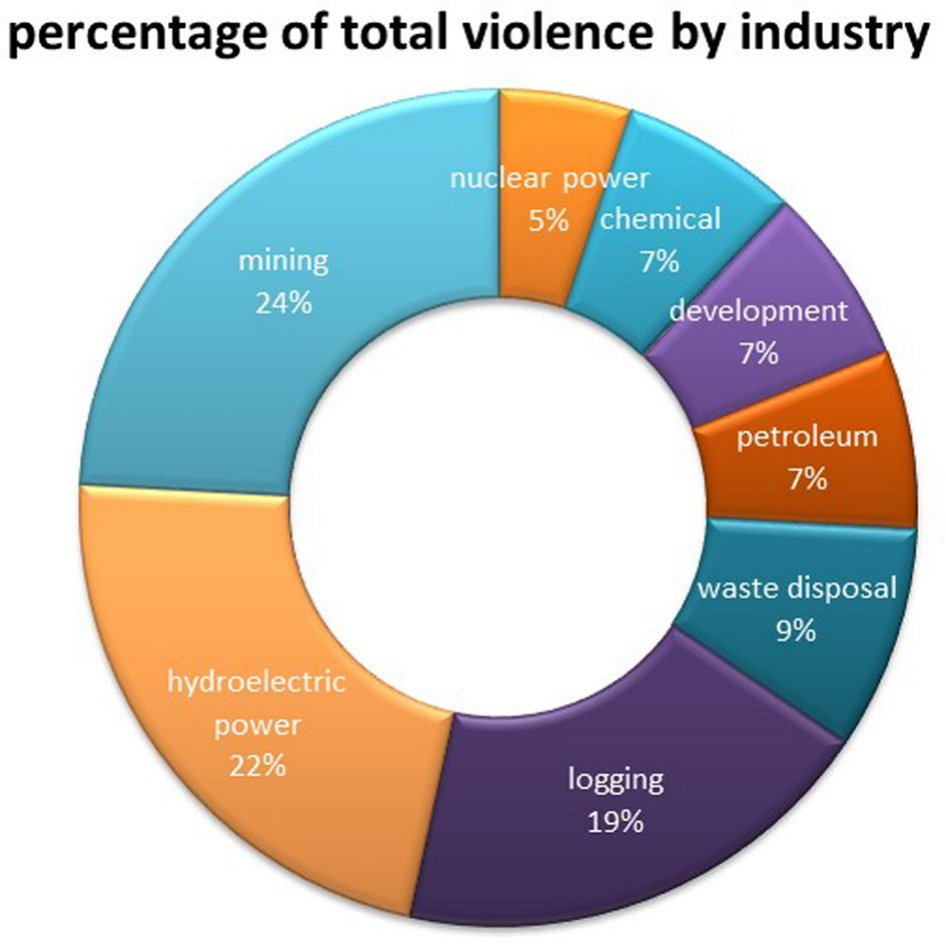
Violence by industry type

The industry type associated with the protest was the third most important factor influencing violent repression. Violent repression was more prevalent in the mining, hydroelectric power, and logging industries (Table 2). Violent repression was most common in the mining industry (15 cases of violence), followed by damming (13 cases of violence), and logging (11 cases of violence) (Fig. 2; Table 2). In contrast, wind energy, nuclear power, chemical plants, and development protests resulted in largely nonviolent demonstrations. Violent repression of protests that included marginalized groups was most prevalent in extractive industries including logging, petroleum, and mining. These findings support scholars who argue that the subject/object of protest matters (Downey et al. 2010; Tarrow 1998; Tilly 1993). In particular, our results indicate that resource extraction-related projects tend to be significantly more violent than other environment-related projects.

**Table 2:** Prevalence of violence and involvement of marginalized groups in grassroots environmental protests by industry

Percent of protests in industry involving…	*Industry*						
	Hydro-power	Logging	Mining	Chemical plant	Nuclear power	Petroleum	Waste disposal
Violent repression	52	55	50	22	20	27	19
Marginal groups	68	90	71	28	0	33	8
Violence against marginalized groups	62	100	86	25	0	75	0
International funding	40	15	64	33	20	67	15

There are several possible reasons for why extractive industries, more than others, tend to be violent. Perhaps, the high level of capital investment raises the stakes for industry and makes them less able to tolerate dissent (Gartner and Regan 1996). Perhaps, extraction industries are more prone to violence for the same reasons that they are prone to corruption—the industry tends to couple monopoly market control without accountability (O’Higgins 2006). Finally, it may be that the isolated geography of resource extraction means that state capacity is less, making violence more possible (Wood 2010; Herreros and Criado 2009). This connection between extractive industries and violence is important because indigenous groups often have contested rights over the land from which the natural resource is being extracted (Inter-American Commission on Human Rights 2010–2011), and ethnic and economic minorities are disproportionately victims when violence occurs (Downey et al. 2010).

**Fig. 3:**
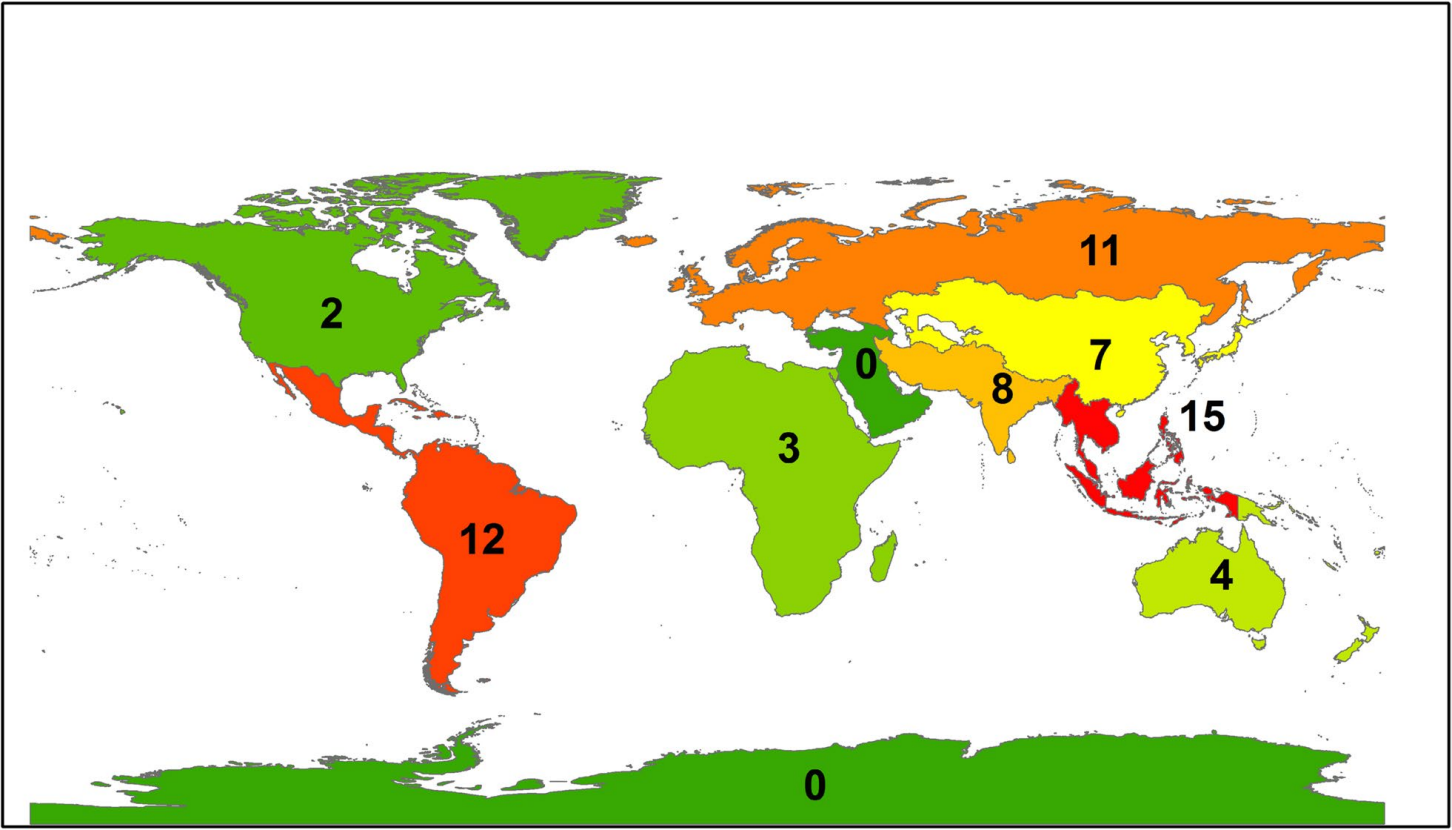
Violent repression of grassroots environmental protests from 1965 to 1980 (n = 175). Geographical regions are categorized according to designations by the United Nations

**Fig. 4:**
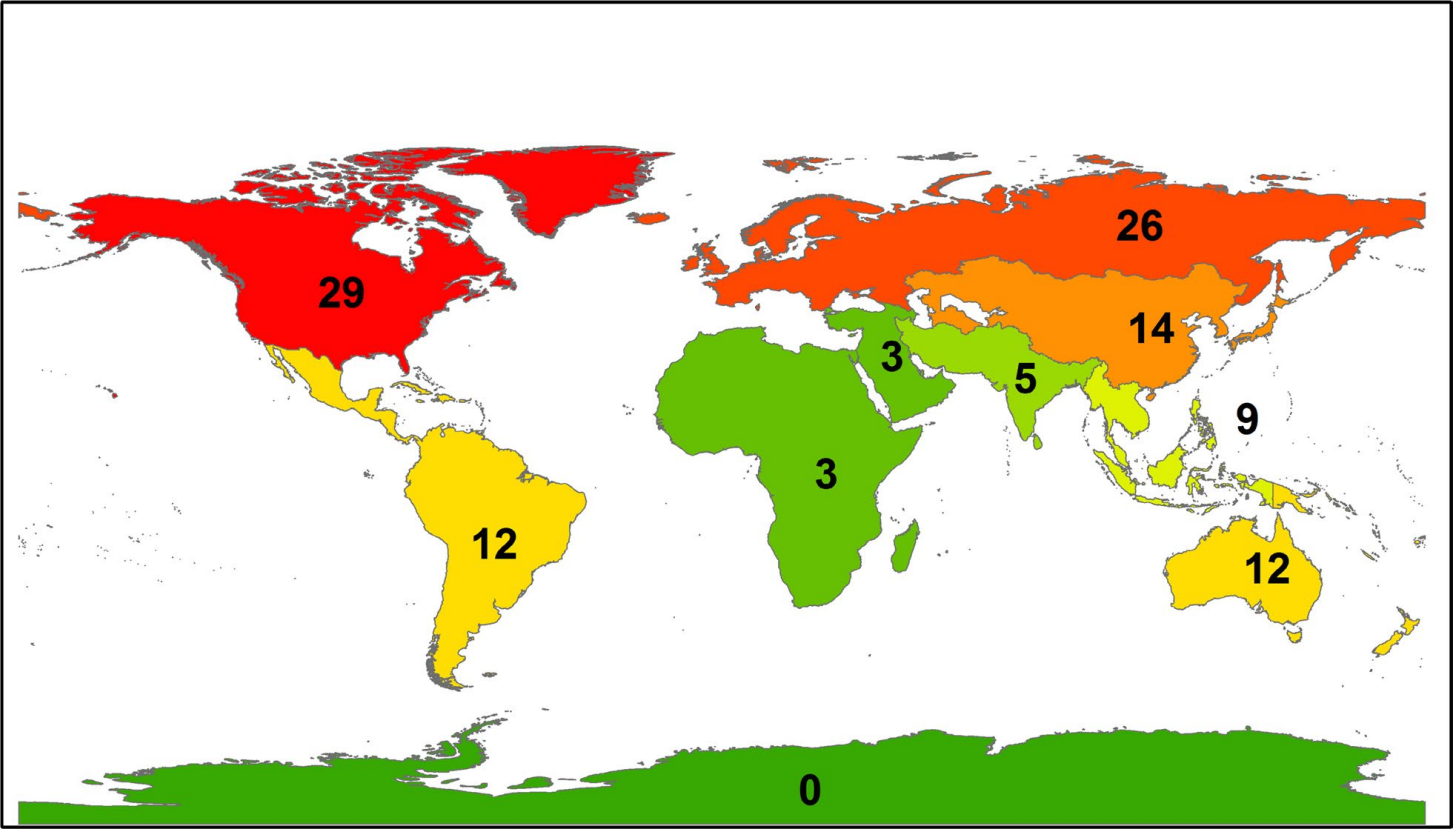
Nonviolent response to grassroots environmental protests from 1965 to 1980 (n = 175). Geographical regions are categorized according to designations by the United Nations

Governmental regime type (free, partly free, or not free) remained an important predictor of violence even when CIRI’s Empowerment Index was included, suggesting that democracy exerts a positive effect on the ability of protests to remain nonviolent, independent of the rights granted to citizens. Examining the data more closely, it becomes clear that, consistent with prior research, democratic countries had much higher overall rates of protest as compared with nondemocratic countries (Poe et al. 1999; Weede 1987; Besley and Persson 2011). Democracies had many more cases of peaceful, nonviolent protests (81 cases) than protests that involved violent repression (31 cases). Nondemocratic countries experienced nearly equal rates of nonviolent responses to (32 cases) and violent repression of (31 cases) peaceful environmental protests. There was a strong geographic component to the violence; violent repression was concentrated in Southeast Asia (6 in free countries, 9 in not and partly free countries) and Latin America (7 in free countries, 5 in not and partly free countries) (Fig. 3), while nonviolent responses to protests was predominant in North America and Europe (Fig. 4).

These findings support the literature that suggests that protests overall are more likely in democracies while violent repression of protests is more likely in nondemocratic states and transitioning democracies (Davenport 1999; Franklin 2009; Chenoweth 2013). These findings also concur with scholars who argue that the external context of a protest is more important than the internal characteristics of the protest organization(s) or their tactics in determining the outcome of the protest (Giugni 2004).

Our findings do not support research arguing that violent tactics by protesters play a significant role in eliciting violent responses from authorities (Porta and Piazza 2007). The large majority (72 %) of violence in the dataset was directed against peaceful protesters who did not respond violently in any way. A minority (14 %) of protests involved violence on both sides (violence against the protesters was significantly larger than the reverse). A tiny minority—only five cases in the whole dataset—were situations where the protesters acted violently toward nonviolent opponents. There may be other context where violent tactics are causally related to violent repression, but in the cases of grassroots environmental protests, the causes of repression appear to have much more to do with the socio-economic context of the conflict and the identity of the aggrieved than protester tactics.

Marginalized groups figured prevalently in environmental protest overall and in protests with violence against activists in particular. Just under half of our cases worldwide involved marginalized groups (45 %). Consistent with the voluminous environmental justice literature, marginal communities were disproportionately targeted as sites for environmentally damaging activities (Bernauer et al. 2013; Homer-Dixon 1994; Koopmans and Rucht 2002). Marginal groups were involved in one-third of the cases of nonviolent responses to protest; a high figure given that the groups usually represented a small fraction of the country’s general population. When examining the involvement of marginalized groups in protests that were violently repressed, the figure rises to 60 %.

Most troubling was the combined effect of industry and marginalized victims. A large majority of the violent repression of environmental protests in the extractive industries of logging, mining, and petroleum involved marginalized groups (100, 86, and 75 %, respectively). Violent repression of dam-related protests also targeted marginal communities, and 68 % of these cases involved marginal groups.

**Fig. 5:**
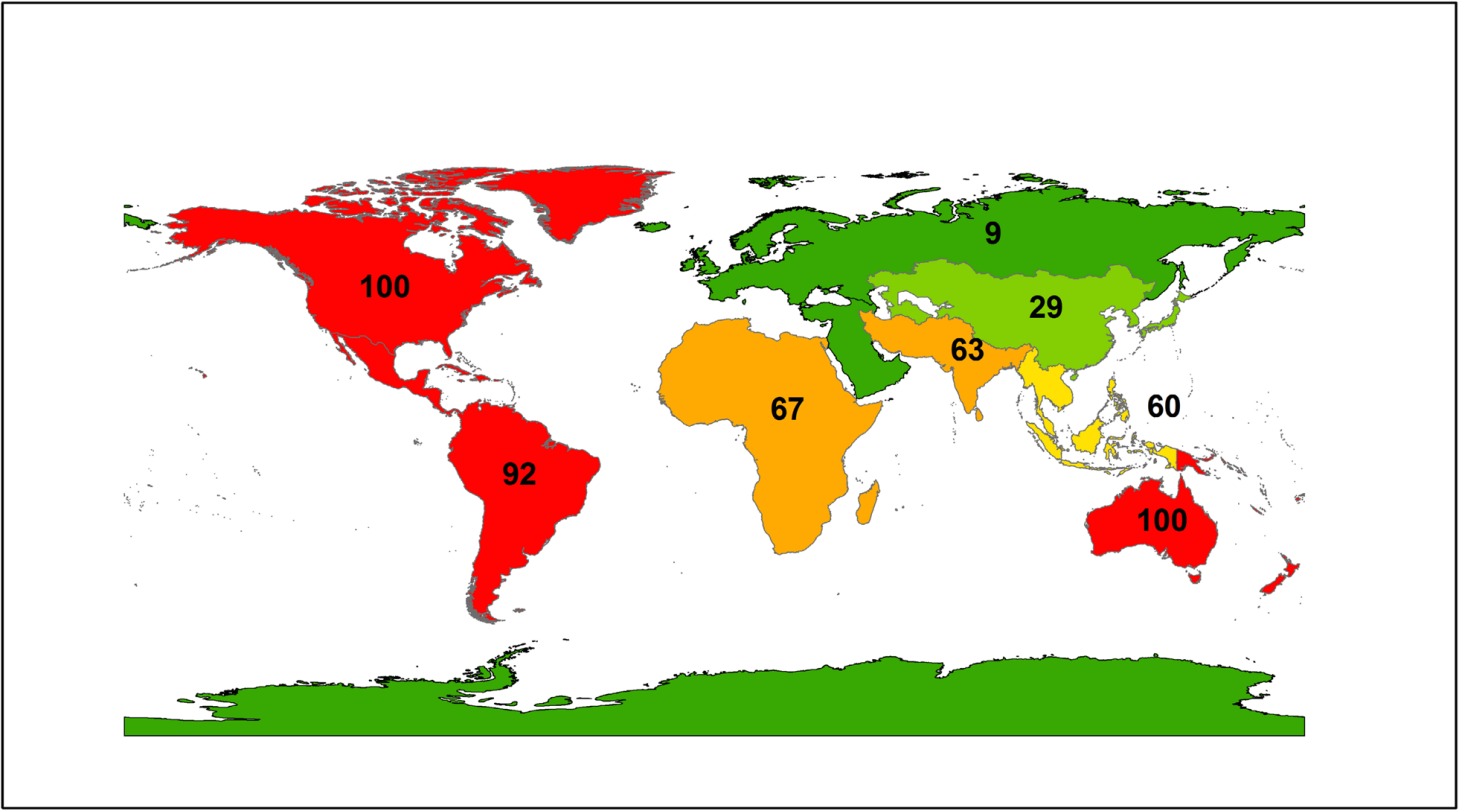
Percent of violent repression that involved marginalized groups

The interaction between regime type and violence against marginal groups was perhaps opposite from what might have been expected by the literature, which suggests that marginal groups are more vulnerable in nondemocratic countries than in democratic ones (Downey et al. 2010; Adeola 2000; Franklin 2009). Although democratic countries overall experienced lower rates of violent repression of protests, those cases where violent repression did occur nearly always targeted marginalized groups (Fig. 5). In the democracies of North America and Oceania in particular, 100 % of violent repression of peaceful environmental protests involved marginalized groups.

This finding refines and complicates the environmental justice literature that holds that marginal groups in poor, nondemocratic countries are the most vulnerable to violent repression (Adeola 2000; Salehyan et al. 2012). Our findings support the vulnerability of marginalized groups in poor and nondemocratic countries—three of the four cases where death tolls exceeded one hundred people occurred in nondemocratic countries; all of them related to extractive industries funded with international funding. However, our findings also suggest that violence against marginal groups engaging in environmental protests is a very serious problem for all countries, irrespective of political regime type. While only one-third of the cases of violence in nondemocratic countries involved marginalized groups (6 of 18 cases), 63 % of violence in free countries involved marginalized groups (17 of 27 cases), and 76 % of violence in partly free countries involved marginalized groups (Fig. 5).

Although international funding was not statistically significant in our model, it was strongly associated with the most extreme cases of violence. In the dataset there were only ten cases where the violence involved more than five deaths, making them relatively rare events, which are difficult to analyze statistically (King and Zeng 2001). In all ten of those cases local people were protesting the extraction of natural resources, and in all but one of the cases international money was supporting the project (Table 3). Furthermore, once again marginalized communities were disproportionately targeted; in nine of the ten cases of extreme violence victims came from marginalized groups.

**Table 3:** Characteristics of protest cases where more than five deaths occurred as a result of the protest

Protest case	Deaths	International funding	Country	Issue	Industry	Geographic region	Marginalized groups	Start year	Duration (years)	Regime type
Belo Monte Dam	6	Alcoa, Vale (mining)	Brazil	Conservation	Hydroelectric power	Latin America	Yes	1987	25	Partly free
PT Inti Indorayon Utama Indonesian Paper Mill	6	No	Indonesia	Pollution	Paper mill	Southeast Asia	No	1993	6	Not free
Tia Maria Copper Mine	6	Southern Copper	Peru	Pollution	Mining	Latin America	Yes	2010	3	Free
Andean Amayapampa Mine	11	Vista Gold	Bolivia	Pollution	Mining	Latin America	Yes	1996	<1	Free
BHP Billiton Nickel Mine in Sibuyan Island	20	BHP Billiton	Philippines	Conservation	Mining	Southeast Asia	Yes	2005	8	Partly free
Xstrata Mine	24	Xstrata	Peru	Conservation	Mining	Latin America	Yes	1985	28	Free
Occidental Petroleum Company Drilling	30	Occidental Petroleum	Colombia	Conservation	Petroleum	Latin America	Yes	1992	13	Partly free
Shell Oil Ogoni Protest	2000	Shell Oil	Nigeria	Pollution	Petroleum	Africa	Yes	1992	21	Not free
Chixoy Dam in Rio Negro	5000	World Bank IADB	Guatemala	Conservation	Hydroelectric power	Latin America	Yes	1982	31	Not free

Nine of the ten cases had funding with international money and nine of the ten cases also involved marginalized groups

In this small subset of cases, where violence resulted in the death of between five and five thousand individuals, violence was extensive and culpability was complex. In most cases, the local government or its agents were the direct perpetuators of the violence, but the disputed project involved significant international funding that influenced the decision to repress the protesters with violence. Some of these cases are well known and well-researched, such as the massacre of Ogoni people in Nigeria in the early 1990s as their protest against petroleum projects in their area expanded into broader demands for self-determination. Other cases that resulted in intermediate numbers of deaths are less well known, such as the 1990 ‘Christmas Massacre’ in Bolivia where 11 miners were killed when government forces gave the miner’s union occupying a tin mine a scant fifteen-minute warning before they open fired.

Although not statistically significant, international funding was disproportionately found in the cases of violent repression across the dataset (it was present in 29 % of the nonviolent cases but 45 % of cases where the protests were violently repressed). Furthermore, when international funding was coupled with an extractive industry, the chances of violence rose dramatically. International funding was involved in a large majority of the projects that elicited violence in the mining (78 %) and petroleum (100 %) industries. Of the industries that spurred primarily nonviolent responses to protests, most of the isolated incidents of violent repression that did occur in these industries also involved international funding: wind energy (100 %), chemical plants (50 %), development projects (50 %), and waste disposal (40 %). These findings support scholarship that argues that extractive industries funded by international capital are prone to violence (Downey et al. 2010). They also suggest that international funding may increase the propensity of violence even in industries that are not extractive and not ordinarily prone to violence.

## Conclusions

This study represents the first comprehensive worldwide assessment of violent repression of grassroots environmental protests. Our findings demonstrate that violence against peaceful environmental protesters is not just a problem in poor, nondemocratic countries. Violence against peaceful environmental protesters occurred most frequently when communities that included marginalized groups protested natural resource extraction in their community.

Previous studies of environmental justice have frequently argued that enhancing the socio-economic standing of victims and potential victims is the best way to combat violence (Roberts and Ash 2009; McGurty 1997; Aldrich 2008; Rabe 1994; Giugni 2004; Ash et al. 2009). Our findings support this common recommendation: disrespect of fundamental human rights and low per capita income were the two most significant factors increasing the likelihood that any given grassroots environmental protest would be violently repressed. However, these two policy recommendations are vague, broad, and difficult to implement.

Our findings that victims of violence are most commonly found in communities containing marginalized groups and the frequent involvement of international funding in cases of violent repression enables us to refine our policy recommendations. We recommend that sustainable development initiatives targeting local communities be built into the project plans for internationally funded development projects, especially when those projects are related to extractive industries. These initiatives would involve integration of local communities into the governance structures present at the site of the project, building economic frameworks that create jobs and economic opportunities for the community during and after the project takes place, and extensive plans for environmental mitigation of any harm caused by the project. These locally focused sustainable development initiatives should be created for all projects, not just for those located in poor countries.

The process of working with local communities to ensure that the development project minimizes its effects on the local population and mitigates its environmental damage, will help create the ‘social license’ necessary for the development to proceed smoothly (Gunningham et al. 2004; Prno and Slocombe 2012). Creating governance structures that engage local stakeholders early and establish clear grievance procedures can reduce the likelihood that local community members will protest a given project and also reduce the likelihood of violence if protests do emerge (Holliday and Yep 2005; Lemos and Agrawal 2006). Industry now has many decades of experience incorporating sustainable development initiatives into large-scale development projects as a component of their risk management and CSR initiatives (Breshears et al. 2005; Castka and Balzarova 2008); http://www.commdev.org). Furthermore, international governmental organizations such as the United Nations and the World Bank have developed comprehensive frameworks for incorporating sustainable development practices into both business development models as well as local and national regulatory frameworks (Ruggie 2008, 2011). The International Finance Corporation’s CommDev (Commdev.org) website has an extensive repository of practical information related to incorporating sustainable development into project plans, including a Financial Valuation Tool, which helps firms and investors estimate the financial return on site-specific sustainability investments. These industry and international organization-developed tools also offer guidelines for appropriate ways to compensate individuals and communities who are displaced by large-scale projects, which is another important element in the efforts to eliminate violence related to these projects.

Responsible corporations and industries are already incorporating local sustainability initiatives into their business models. Industrial groups need to become more active in disseminating the best practice models that they have developed, increasing the pressure on non-compliant firms to raise their standards. Governments, both local and national, should support industry efforts by supporting the projects of corporations that incorporate local sustainable development into their project plans and rejecting projects that do not. Nonprofit and advocacy organizations concerned about human rights and environmental sustainability as well can help support local sustainability initiatives in communities that host extractive industry development projects. Local sustainability should be actively incorporated into large-scale development projects, especially when those projects are taking place in communities that contain marginalized people. Promoting local sustainability as part of development projects will not only enhance environmental outcomes, it will also help prevent tragic human rights abuses that have, unfortunately, been a common outgrowth of extractive industry projects around the world.

## Footnotes

1 For example, the violence against the Ogoni people in Nigeria as a result of their protests against Shell Oil certainly contributed to Nigeria’s high terrorism rankings in the 1990s.
